# TiO_2_ Nanoparticles Are Phototoxic to Marine Phytoplankton

**DOI:** 10.1371/journal.pone.0030321

**Published:** 2012-01-20

**Authors:** Robert J. Miller, Samuel Bennett, Arturo A. Keller, Scott Pease, Hunter S. Lenihan

**Affiliations:** 1 Bren School of Environmental Science and Management, University of California Santa Barbara, Santa Barbara, California, United States of America; 2 Marine Science Institute, University of California Santa Barbara, Santa Barbara, California, United States of America; 3 University of California Center for Environmental Implications of Nanotechnology, University of California Santa Barbara, Santa Barbara, California, United States of America; 4 Department of Environmental and Occupational Health Sciences, University of Washington, Seattle, Washington, United States of America; Argonne National Laboratory, United States of America

## Abstract

Nanoparticulate titanium dioxide (TiO_2_) is highly photoactive, and its function as a photocatalyst drives much of the application demand for TiO_2_. Because TiO_2_ generates reactive oxygen species (ROS) when exposed to ultraviolet radiation (UVR), nanoparticulate TiO_2_ has been used in antibacterial coatings and wastewater disinfection, and has been investigated as an anti-cancer agent. Oxidative stress mediated by photoactive TiO_2_ is the likely mechanism of its toxicity, and experiments demonstrating cytotoxicity of TiO_2_ have used exposure to strong artificial sources of ultraviolet radiation (UVR). *In vivo* tests of TiO_2_ toxicity with aquatic organisms have typically shown low toxicity, and results across studies have been variable. No work has demonstrated that photoactivity causes environmental toxicity of TiO_2_ under natural levels of UVR. Here we show that relatively low levels of ultraviolet light, consistent with those found in nature, can induce toxicity of TiO_2_ nanoparticles to marine phytoplankton, the most important primary producers on Earth. No effect of TiO_2_ on phytoplankton was found in treatments where UV light was blocked. Under low intensity UVR, ROS in seawater increased with increasing nano-TiO_2_ concentration. These increases may lead to increased overall oxidative stress in seawater contaminated by TiO_2_, and cause decreased resiliency of marine ecosystems. Phototoxicity must be considered when evaluating environmental impacts of nanomaterials, many of which are photoactive.

## Introduction

Phytoplankton are the dominant primary producers in marine ecosystems [1], where they are the base of oceanic food webs and a dominant component of the global carbon cycle, as well as other biogeochemical cycles. As abundant small (0.2–200 µm) single or clustered cells with high surface-to-volume ratios suspended in water, phytoplankton have high probability of encountering suspended particles, including pollutants, especially in coastal zones where contaminants are found in highest concentrations. Phytoplankton depend on solar irradiance for photosynthetic carbon fixation, making them more vulnerable to phototoxic impacts than other groups, such as benthic organisms. Information on the impact of emerging contaminants on phytoplankton, and the potential interaction of contaminants with environmental variables such as irradiance is necessary to predict potential impacts on coastal marine food webs and the ecosystems that they support.

Nanomaterials are an important emerging class of contaminants [2], [3], [4], [5], with potentially wide-ranging ecological impacts within marine and estuarine ecosystems, the expected destination of most industrially discharged nanomaterials. [6], [7] World production of nanoparticulate TiO_2_ is an order of magnitude greater than the next most widely produced nanomaterial, ZnO. Estimated environmental concentrations indicate that among the most commonly used nanomaterials, TiO_2_ may reach highest concentrations in surface waters and pose a significant threat to aquatic ecosystems. [8], [9] Nanoparticulate TiO_2_ is often phototoxic to cells *in vitro* and consequently has been used for wastewater disinfection [10], [11] and investigated as an anti-cancer agent. [12] Oxidative stress mediated by photoactive TiO_2_ is the likely mechanism of its toxicity [13], [14], and experiments demonstrating cytotoxicity of TiO_2_ have used exposure to strong artificial sources of ultraviolet radiation (UVR). [13]

Despite the substantial body of evidence demonstrating phototoxicity of TiO_2_, ecotoxicological studies of this material have seldom measured or manipulated natural levels of UV light exposure in experiments. TiO_2_ is a photocatalyst capable of producing highly oxidizing ROS. The absorption of a photon with sufficient energy (3.2 eV for anatase) is the necessary condition for photochemical reactions to proceed at the photocatalyst surface. [14], [15] When TiO_2_ reaches an electronically excited state an electron (e^−^) is promoted from the valence band to the conduction band, generating a hole in the valence band (h^+^). The resulting electron-hole pair can then recombine or migrate to the surface of the particle and may react with H_2_O or OH^−^ to form OH^•^ or can directly oxidize adsorbed species. The electrons may also react with adsorbed molecular oxygen to form O_2_^−•^ ions. [15], [16], [17] In the water column, TiO_2_ may diffuse and adsorb to the surface of phytoplankton where the UV-activated TiO_2_-plankton complex could then participate in a ligand-to-metal charge transfer reaction [14], in which the phytoplankton cell wall is subject to oxidation. Other potential interactions between TiO_2_ and plankton may arise through diffusion of TiO_2_-mediated ROS from the catalyst surface onto the cell wall or into the surrounding media, where it may attack cells or organic compounds.

Our group has recently reported that although ZnO nanoparticles exhibited significant toxicity to marine phytoplankton, TiO_2_ showed little evidence of toxicity; these experiments were performed under standard conditions with artificial lighting. [18] Here we show that exposure to lights simulating sunlight and emitting UV led to ROS production, with toxic effects in three out of four phytoplankton species tested. To test the hypothesis that UV exposure influences toxicity of nano-TiO_2_ to phytoplankton, we designed experiments with two orthogonal treatments: UV exposure (2 levels: exposed, blocked), and TiO_2_ concentration (5 levels: 0, 1, 3, 5, 7 mg L^−1^). The toxicity endpoint measured was population growth rate, using four widespread species of phytoplankton representing three major groups, the diatoms (Phylum: Heterokontophyta, Class: Bacillariophyceae), green algae or chlorophytes (Phylum: Chlorophyta, Class: Chlorophyceae), and the prymnesiophytes (Phylum: Haptophyta, Class: Prymnesiophyceae).

## Results

### Phytoplankton growth

**Figure 1:**
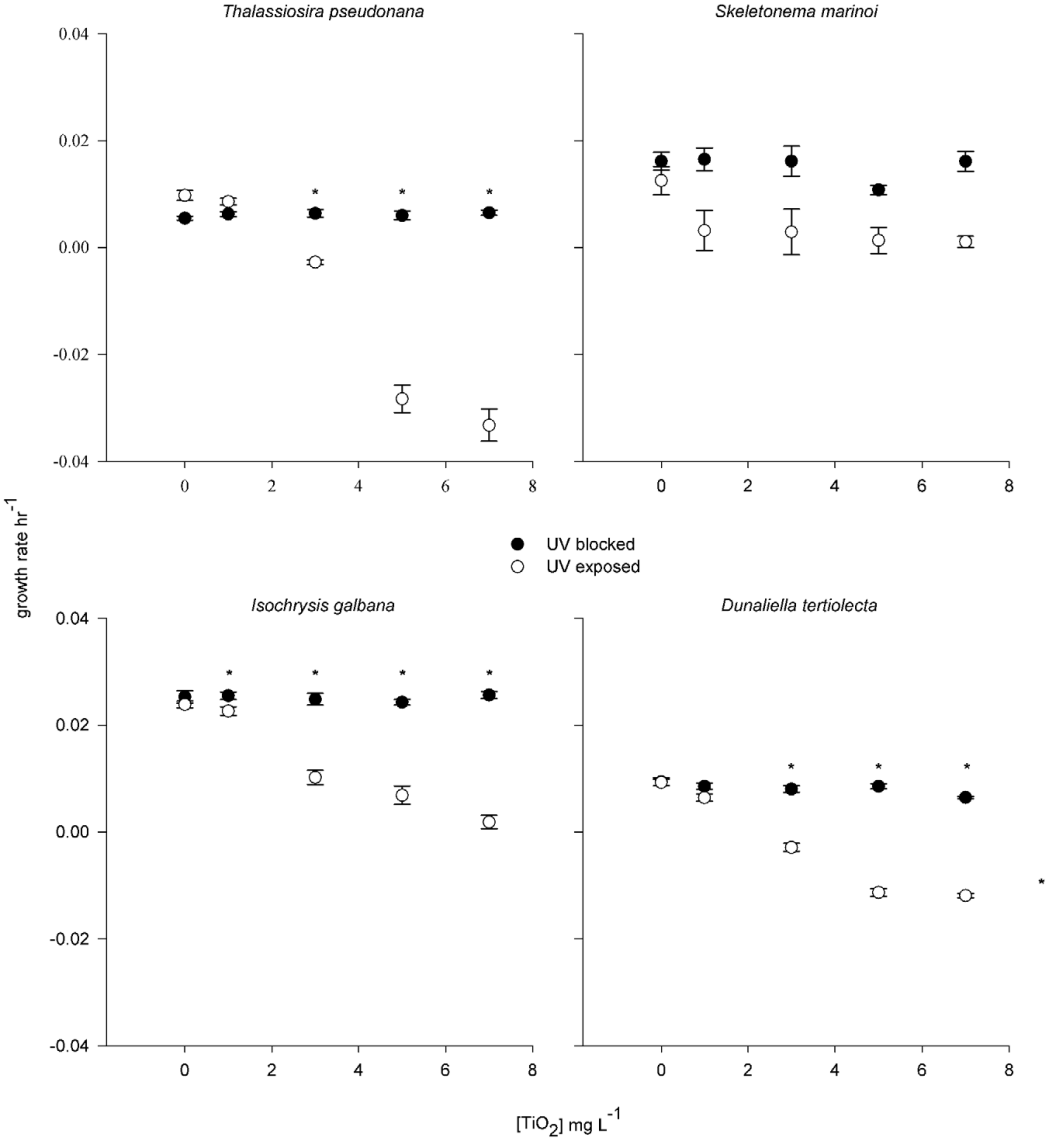
Effect of TiO_2_ nanoparticle (NP) concentration on growth rate of four species of marine phytoplankton, under UV exposure versus UV blocked treatments. Asterisks identify means that are significantly lower than controls (Dunnett's method, *P*≤0.05).

**Figure 2:**
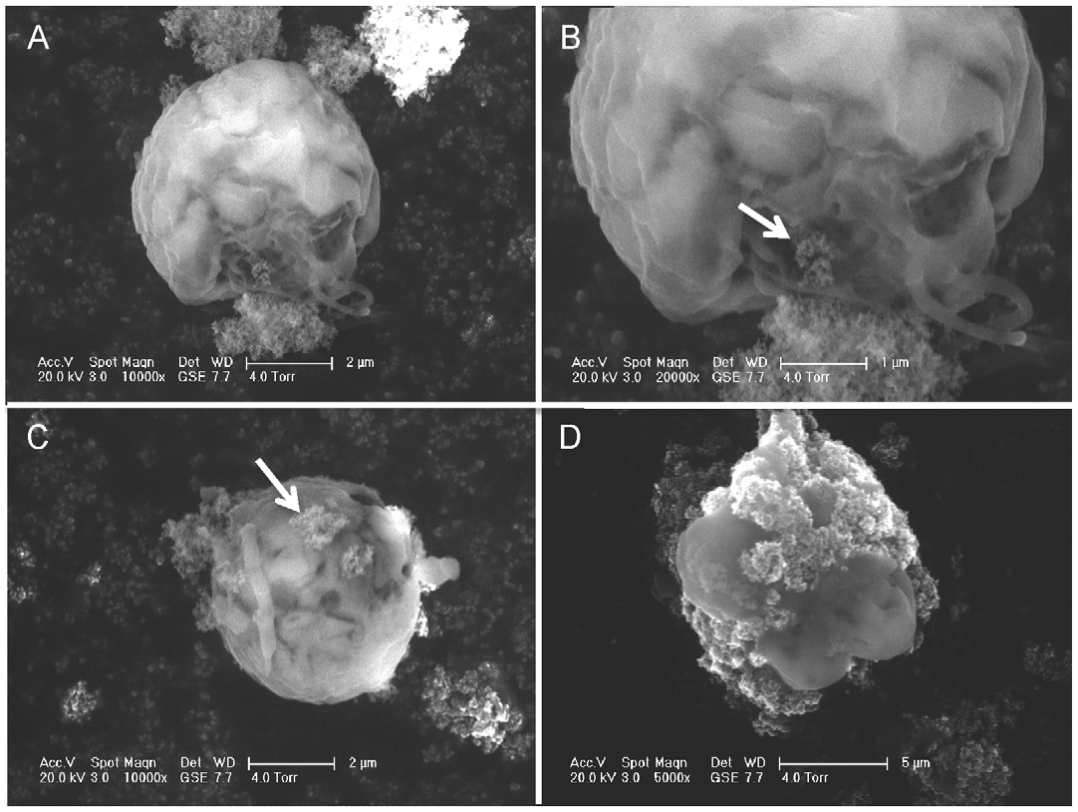
Scanning electron micrographs showing interaction of aggregated nano-TiO_2_ and phytoplankton (*Dunaliella tertiolecta*) cells. Arrows indicate aggregated TiO_2_ particles. Flagellae are visible in panels A–C.

Significant suppression of population growth occurred for three out of four species in the UV-exposed treatment (Fig. 1). In one species, *Isochrysis galbana*, toxicity was evident at the lowest concentration tested, 1 mg L^−1^ (Dunnett's method, d = 2.65, p = 0.02), indicating a no-effect concentration (NOEC) <1 mg L^−1^. In the other two species affected, *Thalassiosira pseudonana*, and *Dunaliella tertiolecta*, significant toxicity was evident at 3 mg L^−1^, although a slight depression of growth rates was seen for *D. tertiolecta* at 1 mg L^−1^ (Fig. 1). No significant effect on growth rates of any species was seen in the blocked-UV treatment except in the case of *I. galbana* at the highest TiO_2_ concentration tested, 7 mg L^−1^. No significant effect of nano-TiO_2_ on growth rate was seen in any treatment for the diatom *Skeletonema costatum*. UVA in the exposed treatment averaged 4.5 (S.E. 0.1, n = 6) W m^−2^ and UVB 4.1 (S.E. 0.2, n = 6) W m^−2^; these levels are comparable to UV intensities near the ocean's surface (<1 m depth in coastal waters). [19] Scanning electron microscopy revealed that TiO_2_ nanoparticles were adhering to the surfaces of phytoplankton cells as aggregations 10's–100's nm in size (Fig. 2).

### ROS production

**Figure 3:**
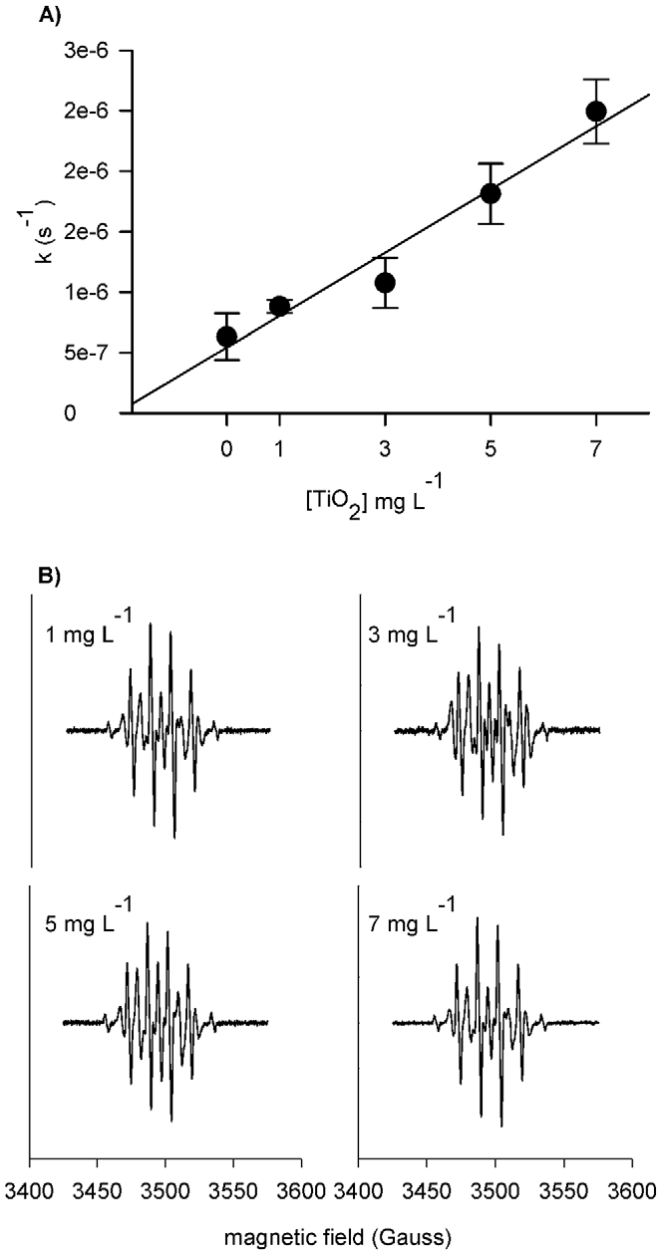
Evidence of OH^•^ production by TiO_2_ exposed to UVR. (A) Photocatalytic production of OH^•^ based on the rate of coumarin degradation. (B) Characteristic 1∶2∶2∶1 EPR spectra with a^N^ = a_β_^H^ = 14.95 of the DMPO-OH spin adduct, produced for all TiO_2_ treatments, confirming the presence of OH^•^. The DMPO-OH adduct was not observed in the absence of TiO_2_.

Production of OH^•^ at low [TiO_2_] in seawater with simulated sunlight, measured using a coumarin probe, was up to 4.6 µM hr^−1^ (±0.26×10^3^ S.E.) at the TiO_2_ concentrations studied (Fig. 3), around 10–20 times higher than natural OH^•^ generation in temperate coastal waters. [20] To confirm the presence of OH^•^, the formation of the Dimethyl-1-pyrroline N-oxide (DMPO)-OH adduct in the presence of UV light was monitored using an *in situ* electroparamagnetic resonance (EPR) spin trap. The DMPO-OH adduct increased over time and with increasing [TiO_2_] (Fig. 3). The characteristic 1∶2∶2∶1 quartet and hyperfine constants a^N^ = a_β_^H^ = 14.95 of the DMPO-OH spin adduct [21] were observed for all [TiO_2_] considered. The EPR spectra were evident after only 20 min of illumination, and coupled with the absorbance and fluorescence data, demonstrate the ability of TiO_2_ to produce OH^•^ in seawater. The experimentally derived steady state [OH^•^] was up to 2.5×10^−15^ M (S.E. 0.255×1.4^−16^), nearly three orders of magnitude higher than that in temperate coastal waters [20].

## Discussion

Our results strongly suggest that photoactivity and UVR exposure need to be considered when designing experiments to evaluate toxicity of photoactive nanomaterials. Previous work has used pre-illuminated TiO_2_ nanoparticles to examine potential phototoxicity to algae and daphnids; the UV light source used was too intense to directly illuminate organisms without mortality. [22] Nano-TiO_2_ that was pre-illuminated in dispersion using a xenon lamp for 30 min at 250 W was more toxic to daphnids than the non-illuminated material, but results were quite variable and no difference was evident for algae. Our results suggest that pre-illumination may not be an appropriate substitute for constant UV exposure in ecotoxicity experiments. Using full-spectrum lighting, as we do here, may reveal toxicity of photoactive nanomaterials where previous results were negative. Halogen lighting was shown to induce a negative effect of TiO_2_ on cell membranes of stream microbes; although UV levels were not measured, the authors asserted that they were environmentally relevant. [23] Although TiO_2_ is the best-studied nanomaterial in terms of its ecotoxicity, little work has been done on algae, and results have varied, although toxicity has generally been relatively low, with effects found at concentrations >10 mg L^−1^. [24] However, these experiments are typically performed under artificial fluorescent lighting that emits little UV. UV exposure has been shown to be necessary for TiO_2_ to act as an antibacterial agent. [25] One study has shown that toxicity of cadmium selenide/zinc selenide quantum dots to the freshwater crustacean *Daphnia magna* was increased with exposure to environmentally relevant levels of UV-B radiation; the cause was explained by both increased release of Cd and ROS generation. [26]

Enriched bacterial growth media has been shown to quench hydroxyl radicals, likely due to nonspecific reactions with organic and nonorganic compounds, leaving only superoxide radicals as the agent of toxicity. [25] The presence of significant quantities of OH^•^ in our experiments shows that natural organic matter in seawater will not eliminate this form of ROS. OH^•^ is the most biologically damaging form of ROS because it attacks all biological molecules in a diffusion-controlled fashion, with a relatively long lifetime of 10^−7^ s and mean diffusion distance of 4.5 nm. OH^•^ also initiates free radical chain reactions, can oxidize membrane lipids, and denatures proteins and nucleic acids. [27], [28] In the oceans, absorption of solar radiation, particularly UVR, by dissolved organic matter in seawater leads to the photochemical production of ROS. [20] These ROS may negatively affect bacteria and phytoplankton by damaging cell membranes or inhibiting photosynthesis. [29] Marine organisms are constantly exposed to some level of oxidative stress, both from external ROS as well as ROS produced by cellular functions such as photosynthesis, and have evolved many ways to deal with this stress, including diverse antioxidant enzymes. [29]

The impact of increasing background ROS levels in marine systems through introduction of nanomaterials may increase the level of oxidative stress on marine organisms and lead to added energetic costs to repair ROS-caused damage, decreasing the resiliency of marine ecosystems to other stresses, including the effects of global climate change. Oxidative stress is one of many stressors experienced by marine organisms, and some, such as thermal stresses, are rising due to climate change. [30] Since phytoplankton are hyperoxic during photosynthesis, they are already exposed to high intracellular ROS concentrations and therefore possess robust antioxidant defenses. [27], [29] Consequently, the impact of TiO_2_ could be even greater on non-photosynthetic organisms, and deserves further attention. ROS-induced stress has been shown to play a role in mass mortalities of fish and other organisms in red tides [31], [32], inhibition of photosynthesis in marine macrophytes [33], [34], loss of vital symbionts in sponges and corals (bleaching) [29], [35], and fertilization success and early development of marine invertebrates. [29] Oxidative stress is already higher in polluted coastal areas. [36] Increases in ROS due to nanomaterials would likely be concentrated around developed coastlines, increasing the already heavy burden of stresses on economically important nearshore ecosystems that support fisheries and recreational activities. These potential impacts should be considered in regulation of nanomaterial discharge and use.

Photoactivity is one of the major useful characteristics of nanoscale TiO_2_, and engineers are continually working to improve the efficiency of photocatalytic activity in this and other nanomaterials. [17] In the case of TiO2, efforts are focused particularly on enhancing photocatalytic activity in sunlight, for applications such as solar energy collection and disinfection. [37], [38] These rapid developments highlight the need to consider the mechanism of toxicity of nanomaterials, and how such mechanisms may change over time. Continual improvement in the photoactive potential of TiO2, for example, suggests that different forms, surface coatings, and dopings of this material will influence toxic effects, and that toxic effects may increase in the future. The fact that different forms of the material will be used for different applications will also influence the environmental transport and fate of the material, and should also be considered in risk analysis.

Our results highlight the need to consider UV exposure in ecotoxicity experiments on nanomaterials with photoactive potential, which includes most metal oxide nanoparticles. The well-documented thinning of the stratospheric ozone (O_3_) layer due to anthropogenic inputs of chlorinated fluorocarbons has caused an increase in UVR reaching the Earth's surface [39], [40], and long-term monitoring has demonstrated complex influences of local atmospheric conditions and global climate change on the amount and variability of UVR reaching the Earth's surface. [40] Interaction of changes in UVR with emerging contaminants could place additional stresses on marine ecosystems in the future, particularly in polar areas where UVR is elevated. [19]

## Methods

**Nanoparticles:** TiO_2_ was acquired from Evonik Degussa Corp. (USA) and was characterized physically and chemically by the University of California Center for Environmental Implications of Nanotechnology (UC CEIN) as standard reference materials for fate and transport and toxicological studies. [41], [42] The TiO_2_ NPs were semi-spherical, 81% anatase, 19% rutile, and 15–30 nm in size. While the primary size of NPs was in the range from 15 to 30 nm, the NPs tend to quickly aggregate in seawater. [42] To produce 10 g L^−1^ stock dispersions, 10 mg of NPs were added to 1 ml of filtered (0.2 µm Millipore) natural seawater, sonicated for 30 min, vortexed briefly, and diluted to 10 mg L^−1^ with filtered natural seawater.

**Phytoplankton:** Four species of phytoplankton were used, *Thalassiosira pseudonana* and *Skeletonema costatum* (centric diatoms, Bacillariophyceae: Centrales); *Dunaliella tertiolecta* (Chlorophyceae: Chlamydomonadales); and *Isochrysis galbana* (Prymnesiophyceae: Isochrysidales). Axenic cultures were obtained from the Provasoli-Guillard National Center for Culture of Marine Phytoplankton (Bigelow Laboratory for Ocean Sciences, West Boothbay Harbor, Maine, USA), and were maintained in standard media (f/2) made with filtered (0.22 µm) natural seawater, which was autoclaved prior to inoculation. To provide inoculant for experiments, algae were incubated under cool white fluorescent lights (14∶10 light∶dark, 100–120 µmol m^−2^ s^−1^) at 20°C with aeration for 5–7 days, until log-phase growth prevailed. Cell densities were measured using a fluorometer as in vivo chlorophyll fluorescence (Trilogy, Turner Designs), which was converted to cell numbers using a standard curve based on counts done with a hemacytometer (Reichert, Buffalo NY). Standard curves were measured at the start of each experiment.

**Phytoplankton exposure experiments:** All experiments were conducted at 20°C, 34 ppt salinity, under the same illumination schedule described above. Fluorescent lighting fixtures fitted with UV-emitting lamps providing simulation of sunlight in the short wavelength region from 295–365 nm (UVA-340, Q-Lab Corp., Cleveland OH) were used for illumination. UV treatment had 2 levels, exposed and blocked. UV levels in the treatments were measured with a broadband radiometer (model UVX, UVP Inc. Upland CA). Blocked replicates were covered with UV-filtering acrylic (Plexiglas G UF-3, Ridout Plastics) that blocked 98% of UV levels measured under the exposed treatment. All glassware was acid-washed, rinsed with purified water (Barnstead nanopure, resistivity >18 MΩ cm), and autoclaved before use. Experiments were run in 125 ml polycarbonate flasks, media volume 50 ml, and were mixed at ∼150 rotations per minute on a rotary shaker (New Brunswick Scientific Co., NJ, USA). NP concentrations tested were 0, 1, 3, 5, 7 mg L^−1^, with five replicates per treatment. Flasks were inoculated with 1–2×10^5^ cells ml^−1^, and cell densities were monitored every 24 hrs for 96 hours.

**Data analysis:** Phytoplankton population growth rates for each replicate flask were estimated as the slope of log-transformed cell count data, obtained through least-squares regression. One-way ANOVA was used to test for an overall effect of NP toxicity on growth rates. Homogeneity of variances was tested with Levene's test; all data conformed to assumptions. When ANOVA revealed significant differences among treatments, *post-hoc* tests were conducted with Dunnett's method, which tests for pairwise differences between each treatment and the control. Statistical analyses were performed using JMP software (Mac vers. 8.0, SAS Institute).

**ROS kinetics:** Hydroxylation transforms coumarin-3-carboxylic-acid (3CCA), into the fluorescent product 7-hydroxy-coumarin-3-carboxylic acid (7OH-3CCA), making this system a sensitive probe for OH^•^ detection.[43], [44] From a stock solution of 10^−2^ M 3CCA (Sigma Aldrich, USA) and 1 g L^−1^ TiO_2_ aliquots were dispensed in Pacific seawater (0.2 µm filtered) to achieve a final concentration of 10^−4^ M 3CCA and 7, 5, 3, 1 and 0 mg L^−1^ TiO_2_ in 200 ml. The 200 ml dispersions were dispensed into polycarbonate bottles and placed on shaker tables. Bottles in triplicate were placed both directly under the UV lights and under filtered UV light (exposed and blocked treatments described above). During the first hour of the experiment, samples were taken every 15 min; subsequently samples were taken daily. After filtering (0.45 µm nylon) samples, [3CCA] was measured using UV-vis spectrometry at 280 nm (Shimadzu Biospec 1601). [7OH-3CCA] over time was used to verify the hydroxylation of 3CCA and to quantify ROS kinetics. The fluorescence data were graphed and the area under the curve was calculated to determine fluorescence intensity. Fluorescence data were then fit with a first-order rate expression and the rate constants were calculated from the characteristic plot. Production of OH^•^ was calculated considering the stoichiometry of coumarin oxidation to 7-hydroxycoumarin by OH^•^ using:
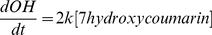
(1)where k is the rate constant in hr^−1^. Mopper and Zhou [20] reported OH^•^ rates of 95.4 nM hr^−1^ for temperate coastal waters and 238 nM hr^−1^ for upwelled coastal water. The rate of OH^•^ production was more than 6 times greater in a seawater system with TiO_2_ present than in coastal waters, ostensibly with high [DOM], the most productive natural photosensitizer in seawater. [20]

The steady state concentration of OH^•^ of coumarin, [OH]_ss_, was calculated using:
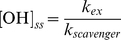
(2)where is k_ex_ is the experimental rate constant from the 7 mg L^−1^ treatment and k_scavenger_ is a scavenging coefficient. [20]

To verify that TiO_2_ catalyzes ROS production in seawater, electroparamagnetic resonance experiments (EPR) were conducted *in situ* using a well- known spin trapping technique. *In situ* EPR is an extremely sensitive technique that allows the direct and indirect detection and determination of ROS kinetics. EPR spin traps are ROS specific, where the first derivative of the absorbance curve provides a unique spectrum generally characteristic of a single ROS. [45]. To 1.8 ml of each TiO_2_ dispersion we added 0.2 mL of 100 µM 5,5-Dimethyl- 1-pyrroline N-oxide (DMPO, Sigma Aldrich, USA). 0.6 ml of the sample was then dispensed into a quartz cell and was placed directly in the EPR (Bruker EMX plus EPR Spectrometer) cavity. A xenon arc lamp (300 W m-2) was used to irradiate the sample through an optical window. Scans were taken every 5 minutes to monitor the EPR intensity.

**Scanning electron microscopy**: Under ambient light conditions, *D. tertiolecta* cells were exposed to 10 mg L^−1^ TiO_2_ for one hour and then centrifuged at 5,000 RPM (Sorvall RC 5B Plus) for 20 min. The supernatant was subsequently removed and the samples were fixed in 6.8 pH phosphate buffered 3% glutaraldehyde for one hour. The cells were washed once with DI water and deposited onto EM stubs with black carbon tape (Carbon Conductive Tabs, 12 mm OD, Ted Pella). Stubs were mounted on the Peltier stage of an FEI Co. XL30 FEG ESEM (Philips Electron Optics, Eindoven, The Netherlands). Imaging was in wet mode at ∼4 Torr, 5°C, using an accelerating voltage of 10 kV. Specimens were not conductively coated prior to imaging. Identity of putative TiO_2_ NPs was confirmed using SEM in combination with energy-dispersive X-ray spectroscopy (FEI XL40 Sirion FEG, Sirion, USA).
